# Microfluidic devices for neutrophil chemotaxis studies

**DOI:** 10.1186/s12967-020-02335-7

**Published:** 2020-04-15

**Authors:** Wenjie Zhao, Haiping Zhao, Mingxiao Li, Chengjun Huang

**Affiliations:** 1 Institute of Microelectronics, Chinese Academy of Sciences, 3 Beitucheng West Road, Beijing, 100029 China; 2 School of Future Technology, University of Chinese Academy of Sciences, Beijing, China; 3 Cerebrovascular Diseases Research Institute, Xuanwu Hospital of Capital Medical University, 45 Changchun Street, Beijing, China

**Keywords:** Microfluidic, Neutrophil chemotaxis, Chemical gradient, Lab on a chip

## Abstract

Neutrophil chemotaxis plays a vital role in human immune system. Compared with traditional cell migration assays, the emergence of microfluidics provides a new research platform of cell chemotaxis study due to the advantages of visualization, precise control of chemical gradient, and small consumption of reagents. A series of microfluidic devices have been fabricated to study the behavior of neutrophils exposed on controlled, stable, and complex profiles of chemical concentration gradients. In addition, microfluidic technology offers a promising way to integrate the other functions, such as cell culture, separation and analysis into a single chip. Therefore, an overview of recent developments in microfluidic-based neutrophil chemotaxis studies is presented. Meanwhile, the strength and drawbacks of these devices are compared.

## Introduction

Chemotaxis, the direct movement of cells along the chemical gradient, is crucial in biologic process, such as innate immunity and cancer metastasis [1, 2]. Neutrophils, which are the most numerous and most important cellular component of the innate immune response, are considered as the first protective barrier against purulent infections [3]. Neutrophils can persist for hours to days within the vasculature until they reach senescence. They accumulate rapidly and efficiently within minutes to areas of inflammation, which depends on the extreme sensitivity of the inciting stimuli. For this effective response, they can detect extracellular chemical gradients and move towards higher concentrations. The migration of these cells is mediated by chemotaxis, which acts as the attractive force to determine the direction in which neutrophils move. Velocity and directional persistence are usually used to characterize neutrophil chemotaxis. The typical velocity is about 1–20 µm/min, which varies with different chemokines and different gradients. The directional persistence means the ratio of relative displacement along the gradient to total path length, which is influenced by the concentration of chemokines [4]. Sensory G-protein-coupled receptors on neutrophil membranes can detect slight concentration change of chemokines. In certain concentration ranges, the neutrophils even response to a concentration difference between across their dimensions of ~ 1% [5]. Mesenchymal stem cells (MSCs) have the potential to differentiate into a wide variety of other cell types. The migration of MSCs was induced by homing signals released by cells at the site of injury and/or inflammation [6]. Chemokines, cytokines, and growth factors (such as IL-6 (interleukin-6) and PDGF (platelet derived growth factor)) released from tissue damage or apoptosis mobilize and recruit stem cells to the damaged site, where they proliferate and differentiate, eventually replacing the damaged tissues [6]. The factors induce upregulation of selectins and activation of integrins on the stem cell surface, enabling cells to interact with the endothelium. Stem cells subsequently adhere and transmigrate across the endothelial layer into tissues. In addition, they are also sensitive to the physical properties of the extracellular matrix (ECM) including stiffness, topography, and dimensionality [7]. Neutrophils are terminally differentiated cells and cannot be genetically manipulated. A model system of neutrophil is the promyelocytic leukemia cells line (HL-60) which can be induced to differentiate into neutrophil-like cells by using dimethyl sulfoxide (DMSO) or dibutyryl-cAMP [8]. The capacity of differentiated HL-60 cells to responds with chemokinesis and chemotaxis to stimuli is different to neutrophils. For example, polymorphonuclear cells (PMN)-like HL-60 cells respond differently to thrombospondin (TSP) than human peripheral blood PMN. HL-60 cells were needed to be differentiated with dimethyl sulfoxide (DMSO), retinoic acid (RA), vitamin D, or l-ascorbic acid (l-AA) before chemokinesis and chemotaxis of HL-cells were assayed [9]. Undifferentiated HL-60 cells did not adhere and were not motile in response to TSP. With differentiation, a maximal response was obtained with 100 to 300 nM TSP, tenfold lower than required for maximal PMN chemotaxis [10]. These differences may reflect (1) an aberration in HL-60 differentiation reflecting their leukemic phenotype (2) differentiation of HL-60 cells to a cell type characteristic of “activated” PMN [10]. The biological mechanism of neutrophil chemotaxis is highly complex, including four steps [11]: neutrophil protection, adhesion to endothelial cells, endothelial cell wall penetration, and migration to infected tissue. Incorrectly signaled neutrophil chemotaxis can cause series cellular malfunctions such as autoimmune diseases and fatal disorders [12]. Therefore, the study of neutrophil chemotaxis is of great significance in clinical medicine.

The classical chemotaxis model is defined as some source releases attractant (or repellent) into the environment, while a distant sink absorbs it, forming a diffusive gradient that can direct cell migration. The signals of chemoattractant were transmitted through interaction with heptahelical G protein-coupled receptors (GPCRs) expressed on cell surfaces. Once the chemoattractant interacts with its receptor on the neutrophil surface, they undergo cytoskeleton rearrangement, cell shape change and polarize. A “leading edge/pseudopod” is showed at the front, which pushes the cell forward and a “trailing end/uropod” is showed at the rear, which enables them to migrate along a concentration gradient [13]. Tweedy et al. [14] proposed an alternative possibility that the cells themselves form a chemical gradient by degrading some ubiquitous local attractant. This self-generated gradient drive cells migration away from their origin. The results showed that self-generated gradients can function over arbitrarily large distances and operate over an exceptionally wide range of ligand concentrations. The breakdown of attractant is absolutely required in self-generated gradients. Breakdown mechanisms include receptor-mediated uptake into degradatory vesicles (growth factor breakdown), dummy or atypical receptors which are used to scavenge ligand or extracellular enzymes secreted or bound to the outer leaflet of the cell membrane. In addition, chemoattractant breakdown is important for sharpening gradients and bringing the attractant levels down below the receptors’ dissociation constant (*K*_*d*_) so they can precisely perceived [15]. Even in common assays, where cells are directly exposed to gradients, chemoattractant breakdown makes chemotaxis markedly more efficient.

Since the concept of chemotaxis was proposed in the 1960s, cell chemotaxis studies have been greatly improved. Traditional techniques in cell chemotaxis researches based on cell culture in vitro are very mature now. One of the most widely used chemotaxis equipment is “Boyden Chamber” [16] developed by Boyden in 1962, which is usually used to detect the chemotaxis of leukocytes and macrophages. However, the Boyden Chamber was unsuitable for observing single cell responses with the unstable chemical gradient profiles, which is also hard to distinguish between chemotaxis and enhanced motility of cells. To observe cell migration in real time, researchers developed “Zigmond Chamber” [5] based on “Boyden Chamber”, which is considered as a prototype device based on microfluidic technology. Zigmond Chamber was the first device to allow direct visualization of cell behavior in the presence of the biomolecule gradient. The main limitation of Zigmond Chamber was that the characteristics of the gradient was determined by the geometry of the device and the diffusion coefficient of the biomolecule. The generated gradient had short life spans (~ 1 h) and was extremely sensitive to evaporation. Then, “Dunn Chamber” [17] and “Insall Chamber” [18] were designed to increase the throughput and achieve long-term observation of cell migration. The gradient generated in the Dunn Chamber was less susceptible to evaporation because source and sink chambers were sealed. However, the gradient cannot be modified once solutions were loaded and the coverslip was secured, like Zigmond Chamber. The Insall Chamber was designed for compatibility with thin coverslips for optimal optical properties and to allow use of high numerical aperture oil immersion objectives. However, a large number of cells would be crushed during assembly and release intracellular factors. The released factors may have chemokinetic or chemotactic activities or synergistic effects in combination with other factors. Therefore, the possibility that the present cell debris has unknown synergistic effects can never be entirely discounted. In addition, traditional devices were obsessed with many issues, such as large number of repeated operations or consumption of reagents. More importantly, these methods have difficulties to reflect the biological characteristics of cells under physiological conditions compared with the complex microenvironment in vivo and microscopic size of cells.

In the recent years, highly controlled chemical gradients have been generated in microfluidic devices under microscale with the development of microfluidic technology [19]. Neutrophil migration and chemotaxis studies based on microfluidic technique have attracted broad interest for its miniaturization and micro-environment control. Compared with the traditional cell chemotaxis devices, microfluidic chips have the unique advantages in cell migration studies as follows: (1) the micron-sized channel is equivalent to the size of cells, which is convenient for precise cell capture and manipulation. (2) Concentration, temperature, pH and other factors can be accurately controlled in the multi-dimensional network channel structure. (3) Lower reagent consumption is required compared with traditional methods. (4) High throughput and high parallelism allow multiple sets of samples to be processed simultaneously. (5) Modularity enhances the possibility of integration. Cell migration, cell capture, cell sorting and other units can be integrated on a single chip. Microfluidic technology provides a new platform for neutrophils chemotaxis studies under the high-controlled gradient conditions. However, it was found that the geometry of the microchannels had a great influence on the cell migration process because mechanical stress, force, and torque on the cell will be amplified as dimensions of the flow chamber are reduced and approach the diameter of the cell [20]. Therefore, the design of the microfluidic device is crucial for microfluidic-based neutrophil chemotaxis studies.

Various designs and strategies of neutrophil chemotaxis microfluidic devices have been developed over the past decade. The initial studies established stable and complex profile gradients in flow-based environment [21–29]. To date, different materials and structures [4, 30–43] have been used to reduce the impact of the flow shear force on neutrophils. In addition, some microfluidic devices [44–46], such as bioinspired microfluidic assay (bMFA) [45, 46], were developed to study the kinetics of leukocyte adhesion [47]. In the recent years, integration devices [26, 27] with the gradual maturity of the gradient generators were fabricated for clinical application [48].

Here, we classified and discussed the structure and working principle of microfluidic devices based on the microfluidic-based neutrophil chemotaxis studies in the past few years.

### Microfluidic technology

Microfluidic is a new biochemical small fluid volume manipulating technology developed from the basis of microelectronics, biotechnology and chemical technology. In 1990s, Manz et al. [49] firstly proposed the concept of micro-total analysis system (µ-TAS) and verified that electrophoretic separation in a µ-TAS was more efficiency than conventional methods. Whiteside et al. proposed a fast template replication method to fabricate microfluidic device by polydimethlsiloxane (PDMS) in 1998, which significantly reduced the manufacturing cost and time. Quake et al. [50] reported a large-scale integrated microfluidic device in 2002. Briefly, microfluidic chip is a miniature biochemical analysis instrument that integrates the experiment steps of biochemical sample pretreatment [51], particle manipulation [52], biochemical reaction [53], detection and result analysis [54] into a device in a scale of tens to hundreds of microns, without sacrificing their performance. Micro biochemical analysis units and systems are built on the microfluidic chips by micro-nano processing technology, enabling rapid detection and analysis of biochemical samples such as organics, inorganics, proteins, and nucleic acids. The advantage of low cost, high-throughput, low sample consumption and miniaturization and integration make microfluidic devices have been widely used in the fields of molecular biology [55], clinical diagnostics [56], and point-of-care systems [57].

The fabrication processes of microfluidic device mainly depend on the selection of materials, the design of the structure, processing, and surface modification. Monocrystalline silicon, quartz, glass, and high molecular polymers are commonly used materials for microfluidic devices fabrication. High-precision two-dimensional or three-dimensional structures can be formed on silicon by photolithography and etching. Quartz and glass have excellent optical properties and are easy to surface-modify. Microstructure, such as metal microelectrodes, can also be processed on quartz or glass, which is similar to silicon. The polymers, such as polymethyl methacrylate (PMMA), polycarbonate (PC), and PDMS, are very suitable for microfluidic devices. PMMA has great light transmission, impact resistance and durability. However, it is difficult for surface modification and has poor biocompatibility and reproducibility during the experiment. PC is a tough thermoplastic resin with good light transmission, heat resistance, impact resistance, and oxidation resistance. It has good mechanical properties in a suitable temperature range, but it is easy to be corroded by organic chemicals. PDMS is the most widely used material for microfluidic devices. It has advantages of prefect light transmittance, strong chemical inertness, non-toxic, low cost, and easy for processing. It also can be modified using polyelectrolyte, surfactant coating layer, and phospholipid bilayer membrane to meet the experimental requirements. Therefore, PDMS is more suitable for microfluidic devices.

Microfluidic experiments generally require microfluidic devices with syringe pumps, microscopes, computer monitors, signal sources, oscilloscopes, etc. However, with the growing maturity of microfluidic technology, it become a new trend to integrate the external analytical instruments onto the chip to meet the needs of portability and automation.

### Neutrophil chemotaxis microfluidic devices

The generation of chemical concentration gradients in the channels of microfluidic devices is vital in neutrophil chemotaxis studies. Based on the gradient generation methods in the channels, microfluidic devices are mainly classified as flow-based microfluidic devices and flow-free microfluidic devices. Flow-based microfluidic devices require a constant flow to establish required gradients, and flow-free microfluidic devices generate gradients based on the chemical diffusion of the chemoattractant. In addition, physical barriers, such as thin microgrooves [30–33], membrane [34–38], or gel [4, 33, 39–43], are used in flow-free microfluidic devices to increase the fluidic resistance to control chemical diffusion. Microgrooves mean micro-sized narrow channels that connected larger-sized microchannels filled with chemoattractant solution and medium. The height and width of microgrooves are about a few micrometers which can reduce the convection of the liquid and generate a stable gradient along the direction of the microgrooves. Gel barrier is 3D network structure that allows diffusion of molecules to generate a stable gradient in a flow-free environment.

### Flow-based microfluidic devices

Flow-based microfluidic devices generate chemical gradients based on the mixing of laminar flow in microfluidic channels [21–29]. Stable gradients perpendicular to the fluid direction are produced by controlled diffusive mixing inside microchannels under conditions of low Reynolds number. A variety of distribution of stable concentration gradients are generated in microchannels by changing channel structures and flow rate.

**Fig. 1:**
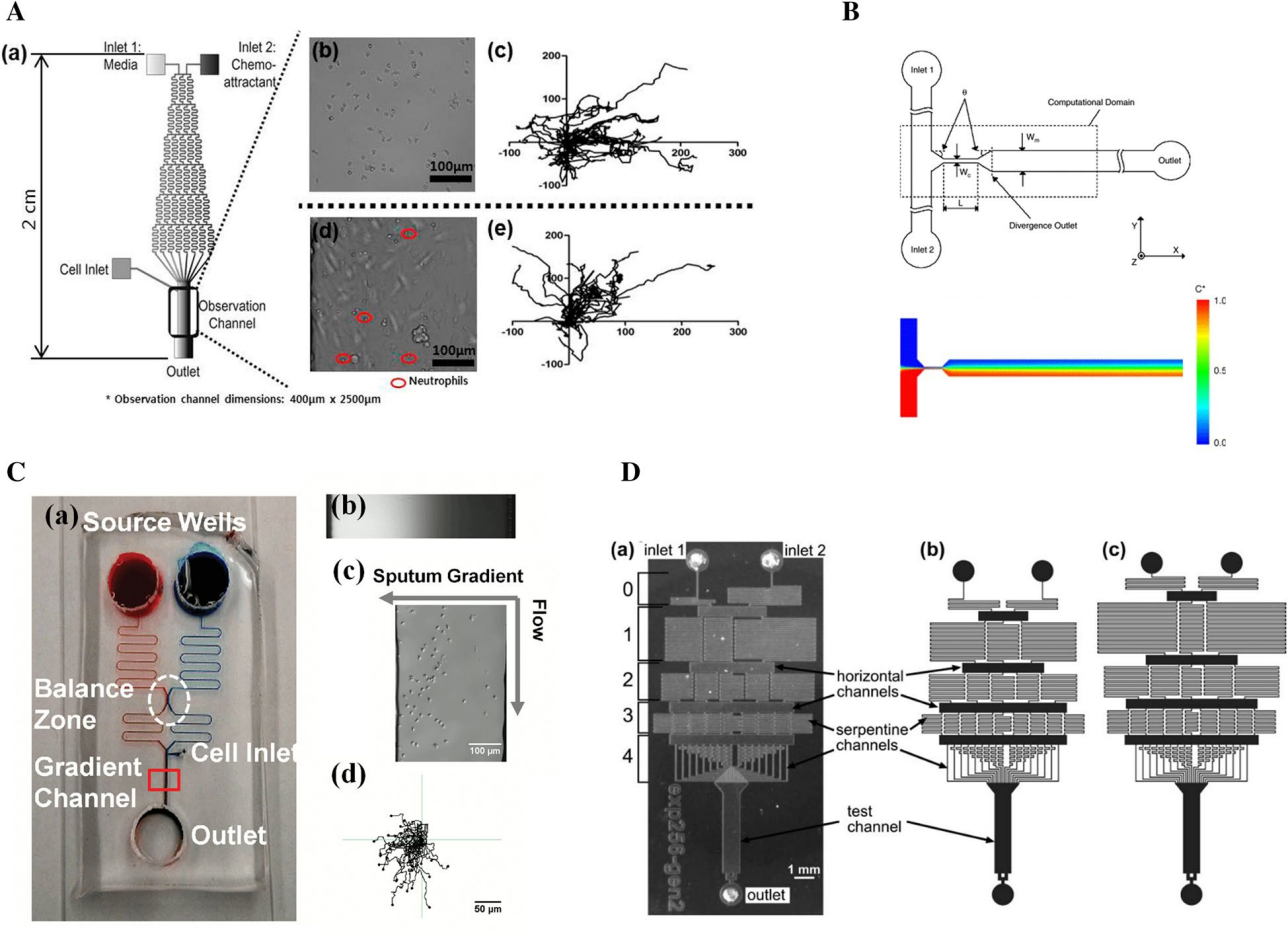
Examples of flow-based microfluidic devices. **A** Multi-mixing, serpentine channel network microfluidic device. A stable concentration gradient was established across the observation channel after multi-stage split recombination in the serpentine channels. (Reprinted with permission from [23]. Copyright (2013) American Chemical Society.); **B** the converging–diverging microchannel to generate desired gradient in a short mixing length based on the enhanced diffusion in the channel. Reprinted from Ref. [29], Copyright (2005), with permission from Elsevier. **C** the microfluidic device with a pressure balance zone unit for stabling the flows from different source inlet wells to generate concentration gradients in the gradient channel. (Figure reproduced from Ref. [25]); **D** the planner microfluidic networks device to establish concentration gradients with any given monotonic function shape. Republished from Ref. [24], copyright 2007, with permission of Royal Society of Chemistry; permission conveyed through Copyright Clearance Center, Inc

Li et al. [22] developed a network of microfluidic channels with multi-stage split recombination. The device generated spatially and temporally linear concentration gradients of IL-8 to study neutrophil chemotaxis. Human neutrophils migration in different IL-8 gradient distribution was test in the microchannel. The result showed that neutrophils exhibit strong directional migration toward increasing concentrations of IL-8 in linear gradients. Kim and colleagues proposed a similar serpentine channel network to investigate how cell–cell interactions influence human neutrophil migration and surface marker expression [23] (Fig. 1A). A stable concentration gradient across the observation channel was established when solutions introduced into the two inlets and mixed while traversing the serpentine channels. This work explored interleukin-exposed neutrophil behavior in a concentration gradient of the bacterial chemoattractant fMLP. Neutrophil migration against a bacterially derived fMLP, with and without pre-activation by interleukins, was evaluated in the presence and absence of endothelial support cells, separately. Grigolato et al. [58] proposed a microfluidic device that consisted of a gradient-generating serpentine network and five observation chambers for fully automated, quantitative assessment of neutrophil chemotaxis. The gradient was established by injecting solutions at the two inlets of the gradient-generating region of the device. The epifluorescence microscope was used to assess the neutrophil migration. This device allowed the precise and reproducible determination of the optimal CXCL2 and CXCL8 concentrations for mouse and human neutrophil chemotaxis. The result showed that IL-4 receptor signaling has inhibition in mouse and human neutrophils migration towards CXCL2 and CXCL8, respectively, and the inhibition is time-dependent.

Mao et al. [28] designed a T-shape microfluidic device to generate concentration gradients in the main channel based on the diffusion of the two fluids introduced from two inlets. Based on this structure, Lee et al. [29] further developed a converging–diverging microchannel to generate a near-constant concentration gradient in electroosmotic flow based on the enhanced diffusive mixing inside the converging–diverging microchannel section (Fig. 1B). Different concentration gradients were established by adjusting the applied electric field and microchannel’s geometry. Compared with the traditional T-shape gradient generator, this device produced desired concentration gradients in a shorter mixing length (shorter by a factor of 2–3.5).

To generate gradient faster, a pressure balance zone was usually used in flow-based devices [25, 26] (Fig. 1C). This design made the downstream gradient generation insensitive to the variation in solution volume of the source inlet wells [25]. Therefore, the requirement of assay operation accuracy was reduced. The structure of the chips with a pressure balance zone was significantly simplified by using zigzag channels before and after the pressure balance zone.

A series of microfluidic networks devices were designed to generate complex concentration profiles and different gradient profiles flexibly [24, 59, 60]. As an example, Campbell et al. [24] designed a planar microfluidic networks device to produce concentration gradients with the shape of any given monotonic function (Fig. 1D). The source solutions were repeatedly split and mixed in a series of k = log2(N−1) stages, and then were combined to create a single stream with the desired shape of concentration profile. Three networks that generated an exponential concentration profile, a linear profile, and a profile with a shape of two branches of a parabola with k = 4 and N = 17 were built and tested. Meanwhile, Hattori et al. [59] reported a serial dilution microfluidic network with a high fluidic-resistance ratio to generate linear concentration profiles and logarithmic concentration profiles spanning 3 and 6 orders of magnitude. Zhou et al. [60] designed a concentration gradient device that established complex profiles of chemical concentration by laterally combining the constituent profiles generated in simple Y-shape or Ψ-shape generator.

The separation of spatial gradients and temporal gradients is a problem in neutrophil chemotaxis studies because the controversy that either the spatial sensing mechanism or the temporal sensing mechanism is principle response for chemical gradient sensing still exists [61]. Neutrophils were exposed under both spatial and temporal concentration changes when they moved in response to a heterogeneous chemical environment. The separation of spatial and temporal stimuli will promote progress towards understanding the mechanisms of cell chemotaxis. Aranyosi et al. [62] built a neutrophil treadmill system that held a moving neutrophil at a specified, unchanging location in a chemical gradient, which decoupled the spatial and temporal gradients around moving cells. The flow within the device was gravity driven with reservoirs of chemokine and buffer and the chemokine diffusion generated concentration gradient. The location of neutrophil within the gradient was changed continuously and precisely to completely restrain the temporal stimulus on the moving neutrophil. This study proved that temporal gradients are necessary for the directional persistence of human neutrophils during chemotaxis.

Flow-based microfluidic devices create gradients through the mixing of laminar flow in microchannels. High-controlled chemical gradient in various distribution profiles can be formed in the channel just by changing the structure of the devices or the flow rate of the liquid. The gradient establishing-time was shorten to a few milliseconds. However, most of the flow-based microfluidic devices require peripheral chemical perfusion and neutrophils are exposed to the constant shear flow since the gradient are established depending on the diffusion across laminar streams. The direction of fluid flow (the direction of neutrophils movement caused by shear forces) is along the channel direction, and the direction of neutrophil chemotaxis is perpendicular to the channel direction. Thus, the movement of neutrophils are affected by shear forces. Most of the autocrine/paracrine factors secreted by neutrophils will be wash away, which may influence the chemotaxis behavior of neutrophils. In addition, the chemical gradient distribution will change along the channel direction, which will also affect the neutrophil chemotaxis studies. The stability of the gradient profiles is limited by the flow rate stability. For this method, sample and reagent consumption cannot be ignored when high flow rates are required.

### Flow-free microfluidic devices

**Fig. 2:**
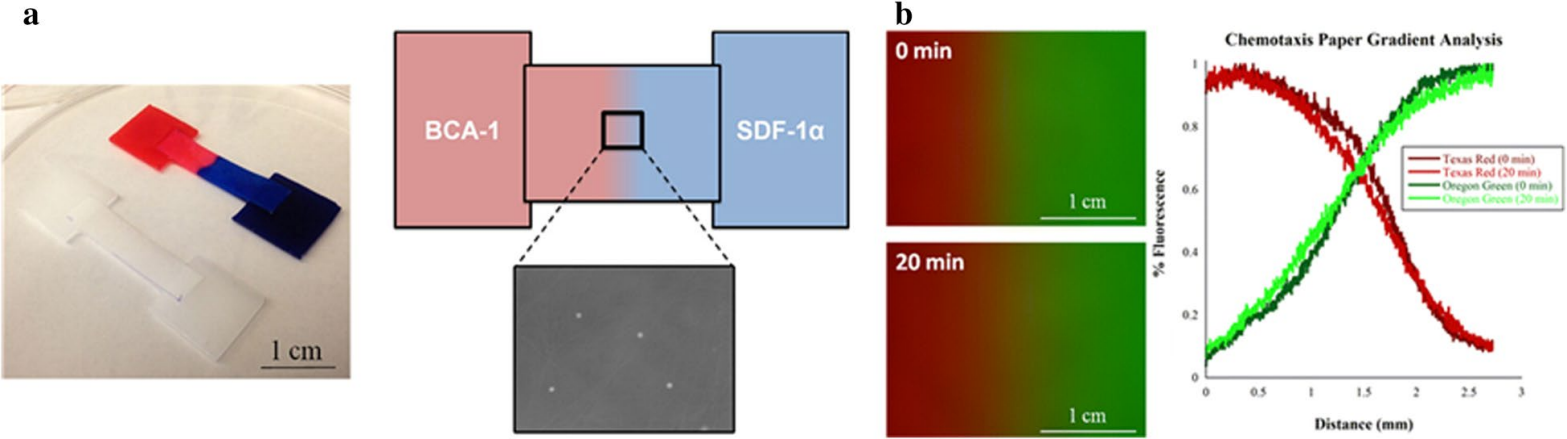
Examples of flow-free microfluidic devices. **a** Gradient generation in paper device; **b** analysis of the paper gradient using fluorescent dextran. False color images of the gradient at 0 and 20 min (left). Normalized pixel intensity analysis over the image from left to right for the gradient at 0 and 20 min (right). Reprinted with permission from [63]. Copyright (2015) American Chemical Society

To overcome disadvantages of flow-based microfluidic devices, flow-free microfluidic devices with chemical concentration gradients in flow-free environment based on the chemical diffusion were developed to reduce the shear forces on neutrophils. For instance, Walsh et al. [63] designed a paper-fluidic device to investigate cell chemotaxis (Fig. 2). All the forces necessary to build competing gradients were provided by the wicking action of paper because when two fluids traversing paper from different direction came in contact, diffusion between fluids established a semi-stable gradient on a time scale suitable for cell chemotaxis. Results showed a stable gradient of chemokine was maintained for 20 min, with a sharp gradient taking place over 2 mm. Thus, cells directly exposed to the gradient were easily to response the change in chemokine concentration. The analysis of the gradient on paper was shown in Fig. 2b. It can be seen that there appeared to be some relaxing of the gradient over the course of 20 min, however, the effect was very minimal. The gradient stretched over 3 mm at the time of zero minutes, and stretch out only on a scale of micrometers over 20 min. Thus, this paper device provided a quasi-steady state gradient over the 3 mm gradient area for the length of the experiment. The proof-of-concept experiment showed significant directed migration of cells to the chemokine gradient over the control condition. Weckmann et al. [64] used a chemotaxis 3D µ-slide (IBIDI) to monitor and quantify neutrophil chemotaxis through combining with simple video microscopic equipment and highly standardized tracking. A stable gradient was generated throughout the channel connected to two chemoattractant-filled chambers. Typical migration profiles of the chemoattactants IL-8, fMLP, and LTB_4_ was identified by using this simplified migration analysis (SiMA) system. To simulate the interstitial spaces and chemoattractant gradients, Boneschansker et al. [65] used microfluidic maze to analyze the migration process of neutrophils. The mazes mimicked the confinement found in interstitial spaces by restricting the migration of neutrophils through channels with 10 × 10 µm cross-section. The mazes were connected to the reservoir that generated a chemokine gradient, which persisted for more than 3 h. Cells in the cell-loading chamber migrated towards the source of chemoattractant through mazes (550 µm in length and 350 µm in width). The result showed different migration patterns of neutrophils for different chemoattractants. For LTB_4_ and fMLP gradients, neutrophils had highly directional migration patterns and moved towards the source of chemoattractant. However, neutrophils had a low-directionality migration pattern and dispersed within mazes, for C5a and IL-8 gradients.

Flow-free microfluidic devices overcome the disadvantage of the flow-based microfluidic devices and generate gradients by free diffusion of chemicals in flow-free environment. Thus, they are less dependent on external controls and the shear forces effect on cells is minimized. However, they tend to generate shallow gradients and are different to create complex gradient profiles. With the development of the flow-free microfluidic devices, they were mainly classified into three types, microgrooves-based devices, membranes-based devices, and gel-based devices. For membranes-based devices and gel-based devices, membranes and gel are used as physical barriers to control chemical diffusion and separate the gradient generation chamber and the fluid flow chamber. Therefore, the shearing effect in the gradient generation chamber is minimum.

### Microgrooves-based devices

**Fig. 3:**
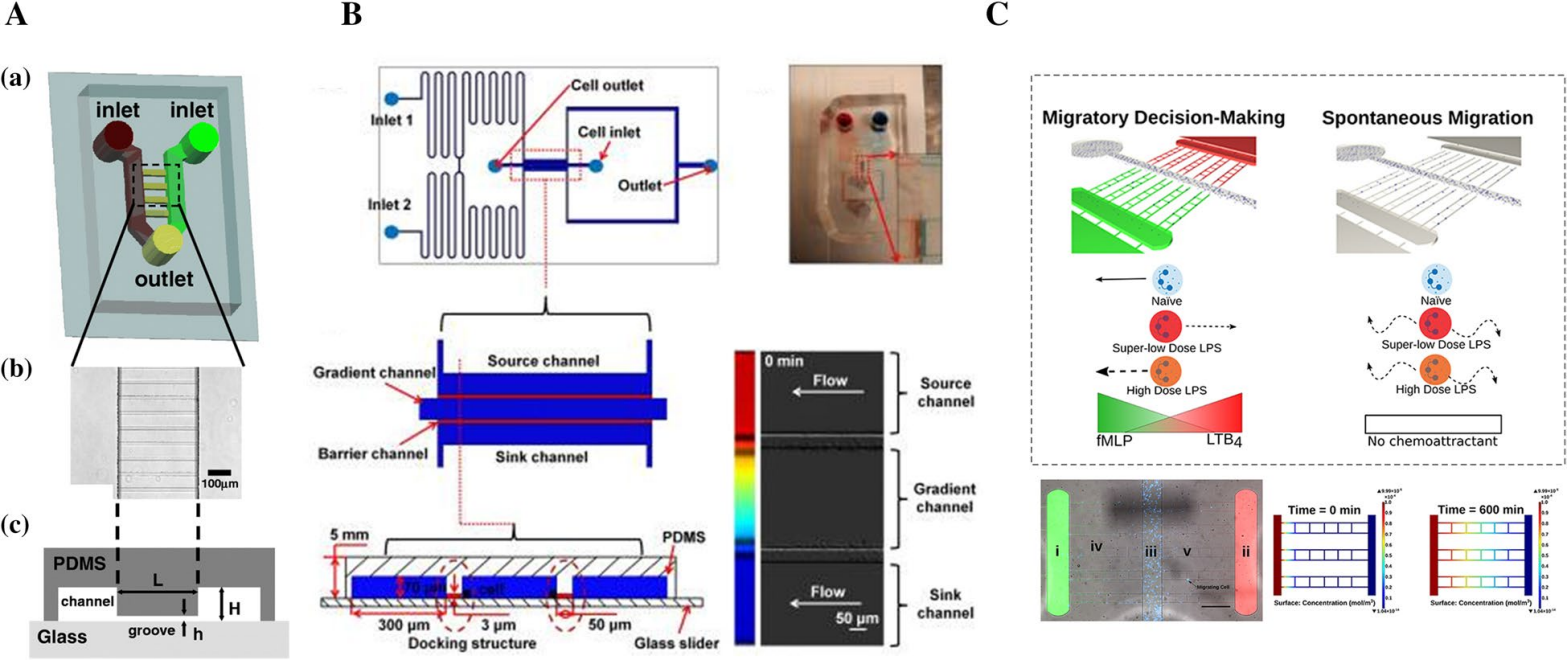
Examples of microgrooves-based devices. **A** Two-channels microgrooves-based microfluidic device. The two high channels connected by a series of thin microgrooves are loaded with different chemicals to generate gradients in microgrooves. (Figure reproduced from Ref. [32]); **B** Illustration of the D^2^-Chip. The gradient channel is connected to the source channel and the sink channel by 3 µm microgrooves defined as the docking structures. The source inlets are loaded with equal volumes of medium with and without chemoattractant, and a stable linear chemoattractant gradient is generated in the middle gradient channel based on the diffusion of the chemoattractant solution and the medium. (Figure reproduced from Ref. [70]); **C** Schematic diagram of the novel microfluidic competitive chemotaxis-chip(µC^3^). Healthy neutrophils(blue), super-low dose (1 ng/mL) of LPS (red neutrophil) and high dose (100 ng/mL) of LPS (orange neutrophil) show different migration under the environment with and without chemoattractant gradients. The gradient was formed within the migration channels from the chemoattractant reservoir to the central loading channel and stayed stable for a long time. (Figure reproduced from Ref. [71])

Microgrooves between channels are a distinct class of flow-free microfluidic gradient generators [30–33, 66]. As shown in Fig. 3A, the “Ladder Chamber”, proposed by Saadi et al. [32], generated the steady state gradients in the microgrooves which connected two parallel channels in flow-free environment. The height and width of microgrooves was 3–10 µm and 10 µm respectively, which were significantly smaller than the two parallel channels (100 µm in height and > 1 µm in width). The fluidic resistance across the microgrooves was high compared to the parallel channels, confining bulk fluid flow in the parallel channels, and minimizing the likelihood of cross-flow through the microgrooves. Therefore, diffusion would be the predominant mode of transport across the microgrooves. Molecules diffused across the microgrooves and generated a gradient when they introduced in one of the parallel channels and buffer in the other. When the system reached steady state, a linear concentration was established. Neutrophil chemotaxis was successfully observed in soluble IL-8 gradient. Irimia et al. [30] developed a similar microfluidic device for precise, passive balancing of flow by contacting two fluid streams before splitting them again and a convection-free, stationary linear or moving steep gradients of chemoattractant was established in an array of microgrooves.

Another type of gradient generators with three main channels are common devices for neutrophil chemotaxis studies [67–70]. The parallel side channels are usually set as the source and sink channel, which are connected to the middle gradient channel through barrier structure such as microgroove channels. The concentration gradients are generated in the middle gradient channel instead of in the microgrooves. The open-chamber “Microjets” device, which was designed by Keenan et al. [67], established a stable and reproducible gradient in the middle chamber within 4 min through fast mixing of jets emitted from the source and the sink channel. The “Microjets Device” was used to develop a method for studying gradient-induced homologous and heterologous neutrophil desensitization [68]. Recently, Yang et al. [70] developed a D^2^-chip which was composed of a middle gradient channel, an outer source channel, and an outer sink channel (Fig. 3B). The source/sink channel were connected to the middle channel by thin barrier channels. Neutrophils were loaded into the middle channel and were pushed towards the channel walls under the pressure difference between the middle channel and the outer channel and aligned along the two sides of the middle channel due to the barrier channels. When the chemoattractant solution and medium reached the source and sink channel, the gradient was generated in the middle gradient channel within 1 min. The D^2^-chip tested neutrophil chemotaxis and the memory effect.

Neutrophil migration process is complex and guided by multi-chemoattractants released from injured tissues. Boribong et al. [71] designed a microfluidic competitive chemotaxis-chip (µC^3^) to measure neutrophil migration in a competitive chemoattractant environment completing chemoattractant gradients in the central channel where cells were exposed (Fig. 3C). The µC^3^ chip was a pump-free stand-alone microfluidic dual gradient device, which consisted of chemoattractant reservoirs for end target chemoattractant (i), chemoattractant reservoirs for intermediary chemoattractant (ii), central loading channel (iii), migration channels (iv), and ladder maze (v). A linear gradient was formed along the length of the migration channels within 15 min, and the completing chemoattractant gradients were generated in the central channel where cells were exposed. It was found that naïve neutrophils migrated toward the primary end target signal in higher percentages than toward the secondary intermediary signal by using fMLP to model an end target chemoattractant and LTB_4_ to model an intermediary chemoattractant. However, the adding of super-low dose LPS significantly increased the percentage of cells migrate toward the intermediary signal. In addition, the result showed that super-low and high levels of LPS stimulation both induced spontaneous neutrophil migration in the absence of chemoattractant gradients.

There were some other microgrooves-based neutrophil chemotaxis microfluidic devices. For example, Hamza et al. [72] designed a U-shape microfluidic device to simulate the biochemical and mechanical confinement condition at sites of injury in tissues and study human neutrophil chemotaxis in response to chemoattractant gradients inside channels. The device was composed of a main channel to load with the chemoattractant solution and neutrophils and a series of U-shape channels to generate gradients. The chemoattractant gradient was established in the U-shape channels by the diffusion between the U-shape channels and the main channel. The concentration was highest at the tip of the U-shape channels and decrease along the two arms of the U-shape. The result showed that more than 90% of neutrophils reversed the direction and migrated persistently after initially moving towards to the higher chemoattractant concentration, and the migration distances away from chemoattractant sources (retrotaxis) were longer than 1000 µm.

Microgrooves-based devices use both convective and diffusive transport to establish a gradient in the cell chamber with no direct shear stresses imposing on the cells [73]. The bulk flow is significantly reduced in the gradient forming channel when the flow rates in the parallel sample and buffer channels are identical. Thus, the effect of shear forces on cells are very small in microgrooves-based devices. Complex gradient profiles can be generated by juxtaposing different designs within a single gradient-generating region [74]. Linear gradients are produced when the width of microgrooves at opposite ends are equal, while nonlinear gradients are produced when the width are unequal. Complex gradient profiles can be established by a serial combination of different microgrooves designs within on gradient generation region. The main limitations of these devices are the requirement of more complicated multi-depth channel fabrication and the limited gradient channel height required for gradient generation. In addition, this kind of devices rely on precisely matching sample and buffer flow rates. Therefore, they are very sensitivity to the mechanical disturbance from the microfluidic system [38].

### Membranes-based devices

**Fig. 4:**
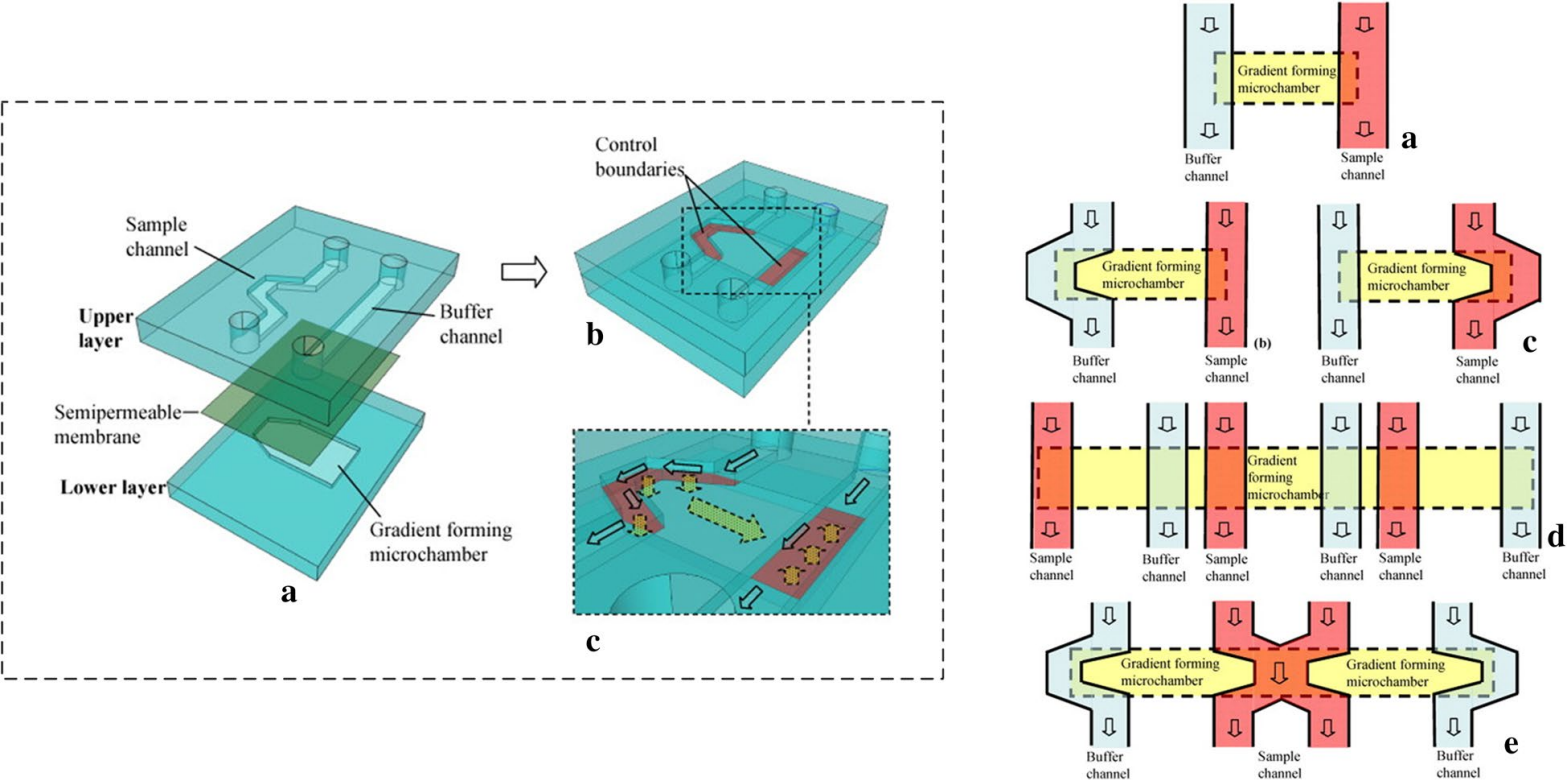
The device configuration of the membrane-based concentration gradient generator. The solid arrows denoted fluidic path, and the dotted arrows denoted diffusion paths. The concentration gradient in the gradient forming channel was controlled to be stable by the running sample and buffer solutions in the respective overlaying channels. Reprinted from Ref. [38], Copyright (2013), with permission from Elsevier

Porous membranes were also used in flow-free microfluidic devices to buffer fluid shear forces and permit the diffusive exchanges of soluble factors between chambers. Thus, porous membranes enabled a vertical alignment of the flow and cell chambers, which was convenient for cell culture and imaging with inverted microscopes [34–38]. Kim et al. and Vandersarl et al. reported microfluidic devices that used porous membranes to separate the generated-gradient from the cell chamber [34, 35]. The flow and cell chambers were allowed aligning in vertical, which was convenient for cell culture and imaging with inverted microscopes. Chung et al. [37] developed a chemotaxis system in which two chambers were separated by a thin (15 nm), transparent and nano-porous silicon membrane that provided effectively no resistance to molecular diffusion between the two chambers to create flow-free chambers in the microfluidic system. The membranes also had excellent optical properties for phase and fluorescence microscopy. The upper chamber provided flow-generated gradient and the lower chamber provided a shear-free environment for cell observation. Shear reduction of more than five orders of magnitude was predict by considering analytical and computational flow models that account for membrane and chamber geometry. Migrating neutrophils were exposed in a chemotactic gradient or fluorescent without any influence from flow. Similarly, Zhou et al. [38] presented a membrane-based flow-free concentration gradient generator (CGG) microfluidic device that included an upper PDMS layer (sample and buffer channels), a bottom layer (a gradient generation microchamber), and a semipermeable membrane sandwiched between two layers (Fig. 4). The CGG device not only maintained a stable concentration through the free diffusion across the membrane but also separated the concentration gradient in the lower layer and the flow of reagent sample and buffer in the upper layer. By adjusting the geometries of channels, concentration gradients with different shapes were generated in the bottom layer. The flow-free environment where concentration gradients generated eliminated the undesirable flow stimulation on neutrophils.

Membranes-based devices couple the channel to the reservoirs through a semipermeable membrane, which eliminates bulk flow while allowing molecular diffusion. Membranes enabled a vertical alignment of the flow and cell chambers, which was convenient for cell culture and imaging with inverted microscopes. This approach is effective in eliminating fluid disturbances in the gradient forming channel. Different shapes of the gradients can be established by changing the geometries of the microchannels and chambers, but the gradient profiles also diminish over an extended period of time [38]. The gradient establishment time is long, which depends on the geometry of the device as well as the size of the molecules [73].

### Gel-based devices

Hydrogel could provide stable diffusion of chemoattractant molecules. The diffusion rate of molecular in gel is slow due to the 3D cross-linked network of gel, which promotes to establish a chemical gradient with long-term stabilization [41]. Therefore, the combination of hydrogel and microfluidic devices is a very common method to generate predictable, reproducible, and long-term stable chemical gradients with high spatiotemporal resolution [4, 33, 39–43].

Cheng et al. [75] developed a hydrogel-based microfluidic device to generate a stable and long-term linear chemical concentration gradient with no through flow in a microfluidic channel. Three parallel microfluidic channels were patterned on a thin piece of agarose gel. Fluid with a constant chemical concentration flowed in one outer channel (the source channel). The fluid with a black buffer flowed in the other outer channel (sink channel). The chemicals diffused across the channels, and a linear chemical concentration gradient was generated in the center channel when the system reached steady state. The chemotactic response of a suspension cell line—*Escherichia coil* RP437 and an adherent cell line—differentiated human promyelocytic leukemia cell line, HL-60 was monitored successfully using this device. Ahmed et al. [76] characterized the hydrogel gradient generators by confocal microscopy and numerical simulation and apply it to chemotaxis experiments with *Escherichia colt* in both linear and nonlinear gradients. The observed cell distribution along the gradients and the established mathematical model showed very good agreement.

Abhyankar et al. [40] proposed a method that provided linear and non-linear soluble factor gradients within a 3D gel matrix by combining variable channel geometries with the principle of infinite sources and sinks. The concentration profiles were maintained for up to 10 days, and the temporally evolving and long-lasting gradients were applied to study the chemotactic responses of human neutrophils and the invasion of metastatic rat mammary adenocarcinoma cells (MtLN3) within 3D collagen matrices.

**Fig. 5:**
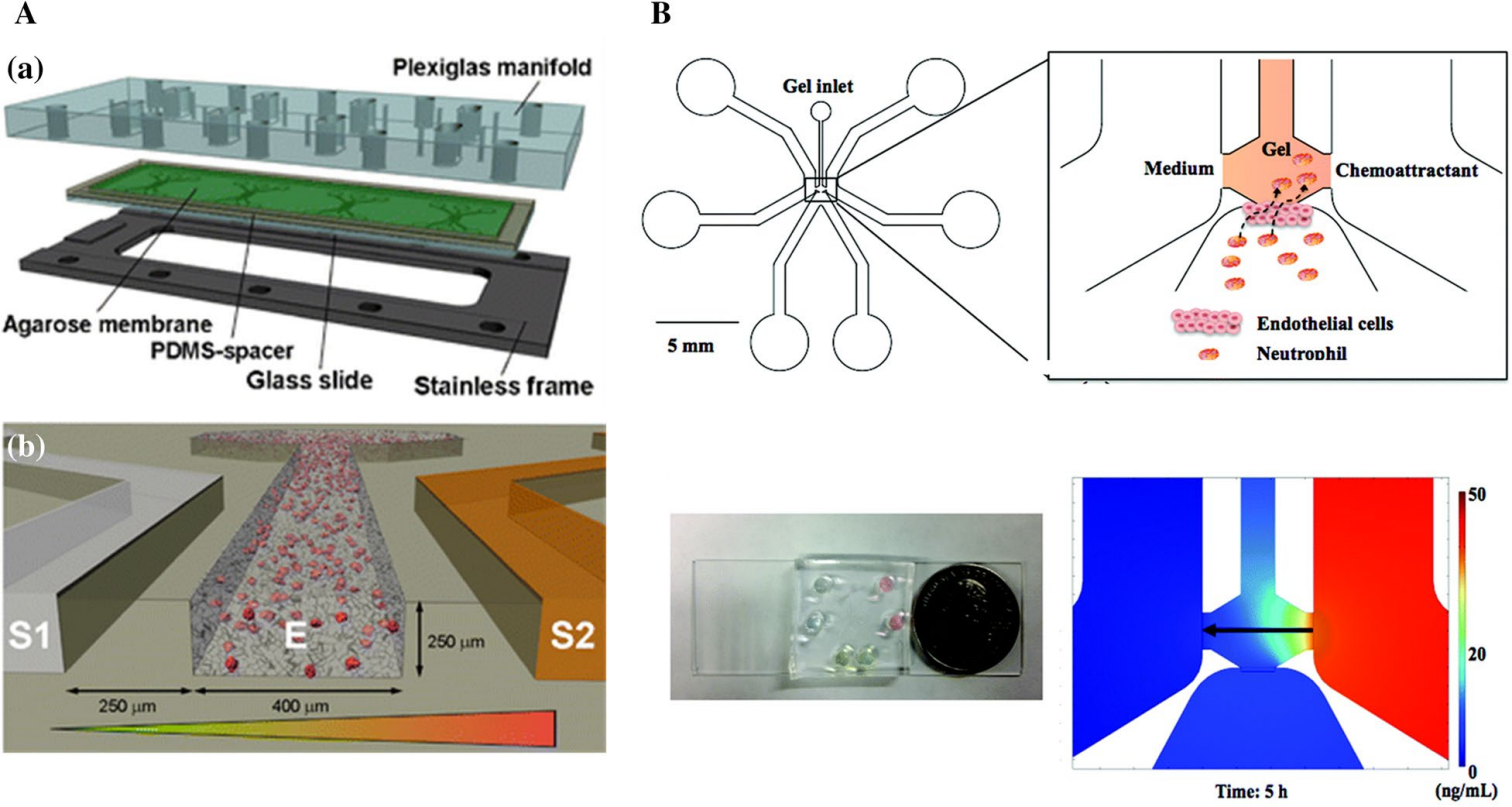
Examples of gel-based devices. **A** The device schematics of the 3D microfluidic chemotaxis device. The device consisted of four three-channel units. Cells and collagen were injected into the center channel together. The chemical gradient was generated in the center channel by introducing media containing different concentration chemoattractant through the two side channels. (Figure reproduced from Ref. [42]); **B** Schematic of gel-based neutrophil TEM microfluidic device. Endothelial cells are cultured on the side wall of the collagen gel, and the chemical gradients are developed by placing the chemoattractant solution or medium on the side channels. Neutrophils will across the endothelial cell layer and move towards the chemoattractant source as the black arrow. Reprinted from Ref. [41], Copyright (2015), with permission from The Royal Society of Chemistry

To eliminate the inherent coupling of the fluid flow and chemical concentration gradients in 3D microfluidic chemotaxis device (µFCD), Haessler et al. [42] presented an agarose-based 3D µFCD to decouple these two important parameters by using an agarose gel wall. It provided the adequate physical barrier for convective fluid flow and protein diffusion at the same time to separate the flow control channels from the cell compartment (Fig. 5A). Petrie et al. [4] used the agarose-based 3D µFCD to study the relationship of the concentration of intermediate chemokines (CCL19 and CXCL12) and the migration of dendritic cells or neutrophils. They found that temporal sensing mechanisms controlled prolonged responses to these ligands.

Wu et al. [41] developed a versatile hydrogel-based microfluidic platform to mimic in vivo neutrophil transendothelial migration (TEM) process (Fig. 5B). Hydrogel provided mechanical support for the growth of an endothelial cell layer in perpendicular direction and highly stable chemical gradients. The results showed that the number of neutrophils migrating across the endothelial cell layer had important relationship with the chemoattractant concentration and the spatial profile of the chemical gradient.

Gel-based devices eliminate flow disturbance in the gradient forming channel through hydrogel, which provide sample molecules diffuse. They are able to maintain temporally non-diminishing gradient profiles with constant replenishment of sample and buffer. Complex concentration gradients profiles could be generated by design different gradient forming channel shape. However, this method needs long generating times (about a few hours) for the concentration gradients due to the slow molecular diffusion in hydrogel [38]. In addition, the optical transparency of hydrogels is relatively poor compared to PDMS or glass, which hinders phase-contrast microscopy [77]. Further improvement and innovation are required to enable more flexible control of gradient generation.

### Integrated neutrophil chemotaxis devices

#### Combined with cell culture unit

**Fig. 6:**
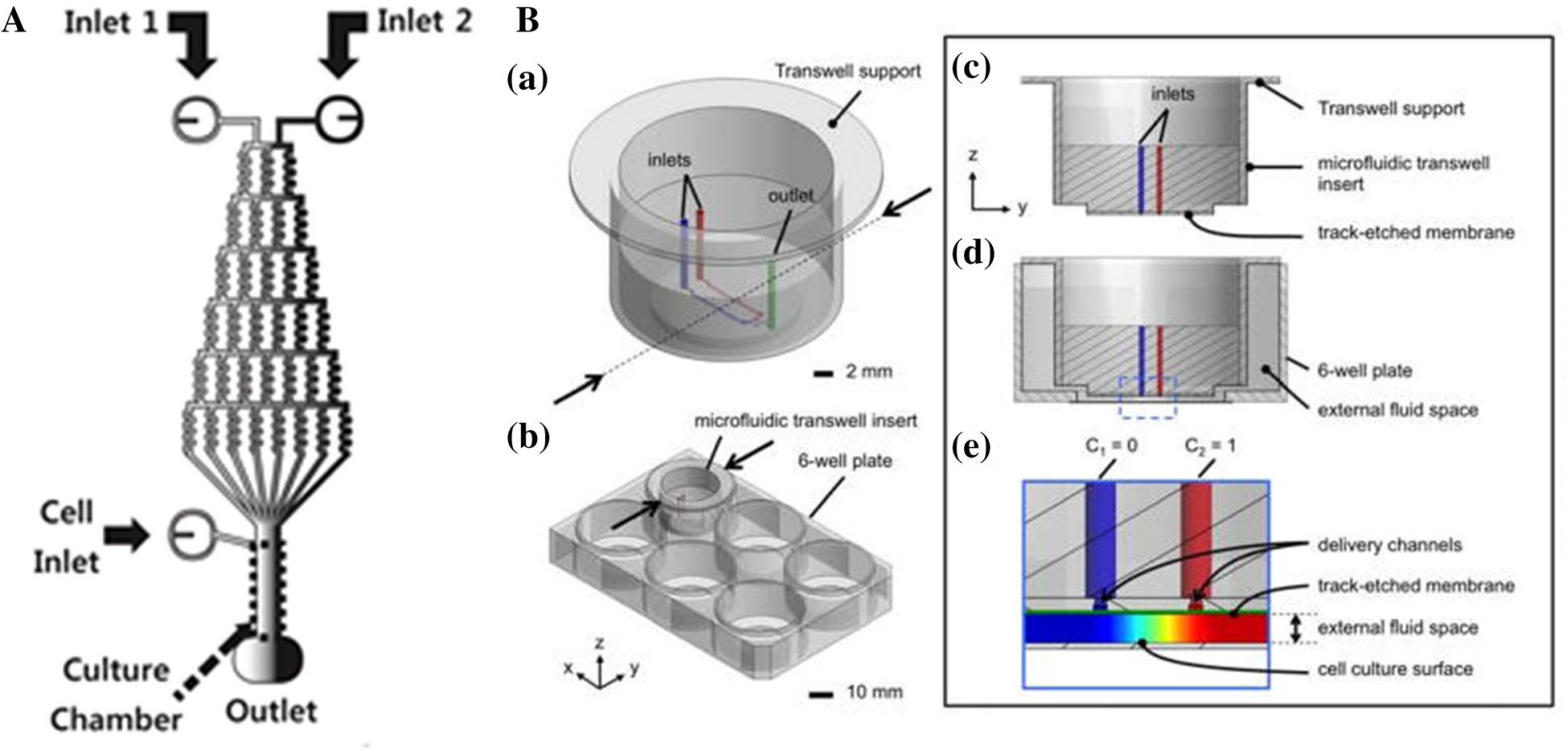
Examples of integrated neutrophil chemotaxis devices combined with cell culture units. **A** Microfluidic device that combines multiple networks and cell culture chamber where the stable gradient is generated. Reprinted with permission from Ref. [80], copyright (2012) American Chemical Society; **B** schematic diagram of microfluidic transwell insert. The device hangs self-supporting in a 6-well. The concentration gradient is generated in the external fluid space (color map) between the track-etched membrane (green line) and the cell culture surface that is approximately 250 µm in height. Reprinted from Ref. [78], Copyright (2014), with permission from The Royal Society of Chemistry, permission conveyed through Copyright Clearance Center, Inc

In most of the single-function microfluidic neutrophil chemotaxis devices mentioned above, cells were injected into the microchannels because long-term cell culture in microchannels is challenging due to shear sensitivity, especially for sensitive cells [78]. With the development of the shear-free environment, some researchers aimed to combine the gradient generation unit and the cell culture unit on the same chip [31, 36, 78–81]. Joanne et al. [79] proposed a microfluidic-based turning-assay chip that consisted of gradient generating networks and cell seeding channels. The device generated precise and complex composite gradients to mimic the conditions the growth cones realistically counter in vivo and study how neuronal growth cones migrate in response to complex combinatorial gradients of diverse external cues. Kim et al. [36] designed a microfluidic device for cell culture and chemotaxis studies. Vertical membranes formed by in situ fabrication were used to avoid fluid flow inside the cell observation chamber. Neutrophils were introduced in the observation chamber and incubated for 30 min, then the mixture of IL-8 and fMLP was introduced in the source chamber. Successful migration of neutrophils up to the concentration gradient of IL-8 was exhibited by experiment. Over 91.7% of neutrophils migrated toward the higher concentration, and the longest distance of the neutrophils travelled in 25 min was 162.5 µm toward the source. The average rate in the x direction and in the y direction was 3.44 µm/min and 0.25 µm/min, respectively. Zhang et al. fabricated a dual-functional microfluidic chip based on rolling circle amplification for cell culture and online IL-8 detection, and successfully applied to analyze the secreted IL-8 in endothelial cells [31]. To study the dynamics of neutrophil chemotaxis under competing chemoattractant gradient, Kim et al. [80] proposed a microfluidic platform, which established a stable and dynamic gradient of chemoattractant across the cell culture chamber (Fig. 6A). Human neutrophils were exposed in competing gradients of four different chemoattractants (leukotriene B4, chemokine C-X-C motif ligands 2 and 8, and fMLP). Over 60% of neutrophils moved toward the stronger signal and the results showed a hierarchy among these chemoattractants of leukotriene B4, chemokine C-X-C motif ligands 2 and 8, and fMLP. Sip et al. [78] reported a microfluidic transwell insert which was compatible with conventional cell cultures and with tissue explant cultures (Fig. 6B). The device hanged self-supported at a distance of ~ 250 µm above the cell culture surface by simply inserting into a standard 6-well plate. The microflows with stable and quantifiable concentration gradients generated by the device moved the integrated track-etched porous membrane, then entered into the cell culture well. This device demonstrated long-term stability of gradient profiles over a large area of approximately 25 mm square and realized the function of analysis the chemotaxis of a large number of cells through direct visualization and automated image analysis. An fMLP gradient was applied to a large population of HL-60 cells (a neutrophil cell line) by using this device. From the total 282 cells observed in the gradient, over 74% of the cells (208 cells) moving towards the gradient, which was the result of quantification by the chemotactic response with an automated tracking algorithm.

The combination of chemotaxis devices and cell culture unit reduce the interruption of cell–cell communication caused by the fluid flow and convection. This method will provide more convenient ways to investigate more cell migration behaviors, such as aggregation and dispersion under determinable concentration gradients while retaining paracrine signaling [36].

#### Combined with isolation unit

Most mentioned neutrophil chemotaxis experiments require purified neutrophil samples. However, the process of purification needs a long time and may cause damage to neutrophils. To remove the purification process such as ultracentrifugation, Mathias et al. proposed a method that stimulated whole blood from umbilical cord blood (n = 6) and healthy control subjects (n = 6) for 24 h with 100ngs/mL of LPS and subsequently isolated CD66b+ with microfluidic technology [82, 83]. The result showed that LPS stimulated whole blood from umbilical cord blood (UCB) demonstrated significant differences in both ex vivo cytokine production and PMN gene expression.

To further simplify the experimental process and reduce the damage caused to the neutrophils by the purification process, some researchers aim to integrate separating units and chemotaxis study units in the same chip [26, 27, 48, 84–94]. Therefore, the integrated microfluidic devices for neutrophil chemotaxis analysis directly from whole blood have been becoming a growing trend.

**Fig. 7:**
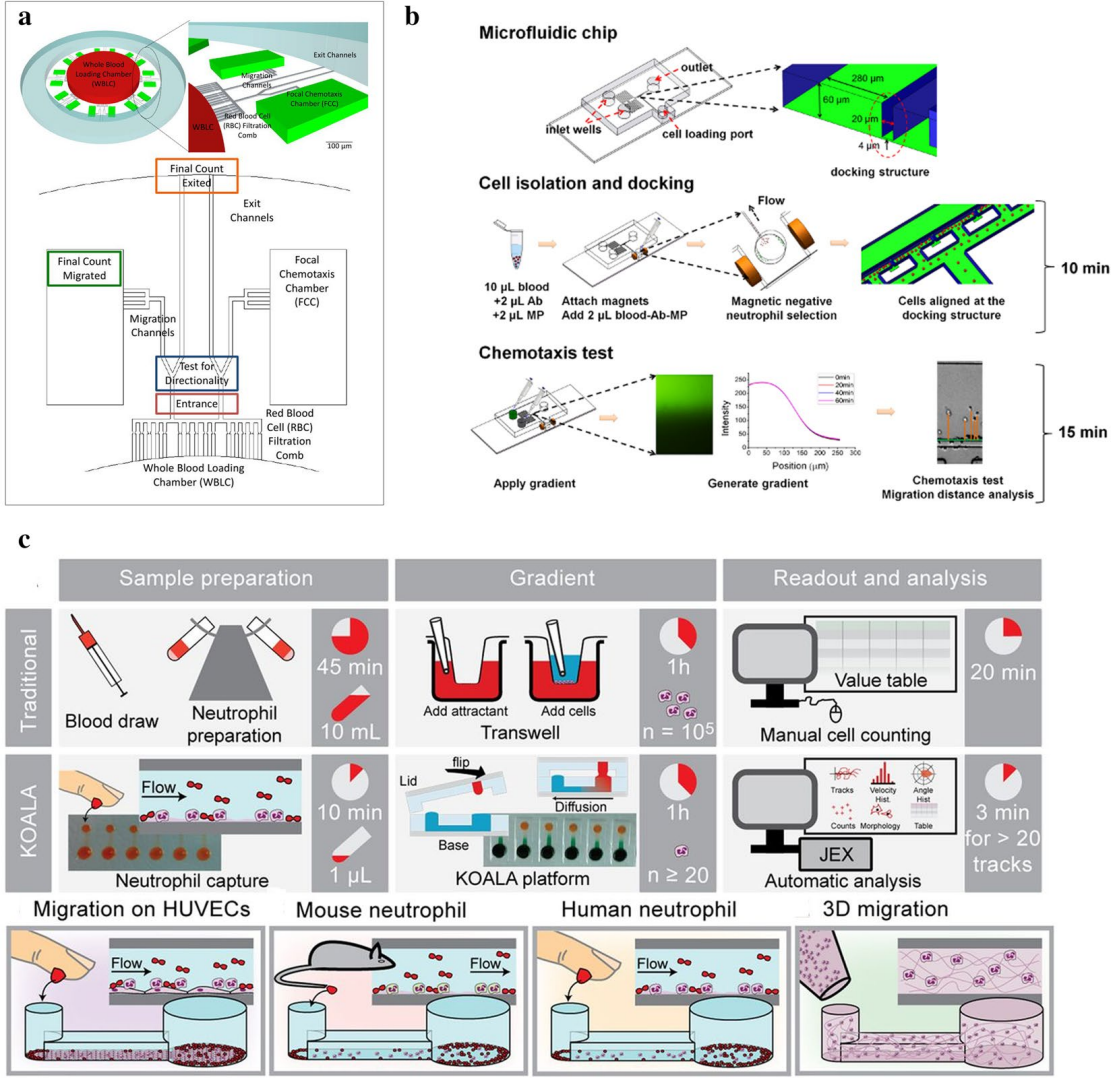
Examples of integrated neutrophil chemotaxis devices combined with isolation units. **a** The donut-shape microfluidic device. Chemoattractant is introduced into the focal chemotaxis chambers (FCCs), and a gradient is generated along the migration channels towards the FCCs. The whole blood is put into the whole blood loading chamber (WBLC). The red blood cells are prevented by the RBC filtration comb and only neutrophils can migrate out of the whole blood and accumulate in the FCCs. This is adapted from Jones, C. N., Hoang, A. N., Dimisko, L., Hamza, B., Martel, J., Irimia, D. Microfluidic Platform for Measuring Neutrophil Chemotaxis from Unprocessed Whole Blood. J. Vis. Exp. (88), e51215, 10.3791/51215 (2014). **b** The all-on-chip device for neutrophil chemotaxis analysis. The device consists of the cell docking barrier channel (4 µm) and the gradient generation channel (60 µm). This is adapted from Yang, K., Wu, J., Zhu, L., Liu, Y., Zhang, M., Lin, F. An All-on-chip Method for Rapid Neutrophil Chemotaxis Analysis Directly from a Drop of Blood. *J. Vis. Exp.* (124), e55615, 10.3791/55615 (2017); **c** Overview of the KOALA platform. Compared with the traditional methods like transwell assay that take many hours to complete all the processes, this platform significantly reduces the experiment time. (Figure reproduced from Ref. [43])

As one example in this direction, Irimia et al. designed a donut-shaped structure (Fig. 7a) to measure neutrophil chemotaxis from whole blood by designing implemented mechanical filters with right angle to selectively block the advance of red cells and made a series of improvement and application [87–92, 94]. The device consisted of a whole blood loading chamber (WBLC) and a series of focal chemotaxis chambers (FCCs) filled with chemoattractant. The generation of the chemotactic gradients was based on the diffusion from the FCCs in the absence of convection [87]. Neutrophils migrated directly from the blood droplet, through small channels, towards the source of chemoattractant due to the ability to deform actively during chemotaxis through microscale channels that block the advance of other blood cells [91]. Jones et al. [87] showed that phlogistic and nonphlogistic cell recruitment were distinguish by integrating an elastase assay into the FCCs. The chemoattractant gradient along the migration channel to the cell loading chamber was established within 15 min and decreased by less than 10% at 6 h. Monocytes and neutrophils induced by LTB_4_ showed significant difference in dynamics. Neutrophils moved through channels at ~ 18 µm/min and reached the FCC in less than 30 min, while the velocity of monocytes was about 5 µm/min and the time of reached the FCC was more than 90 min. The level of elastase produced in real time by monocytes accumulating in the nano-liter chambers was measured to distinguish between phlogistic and nonphlogistic recruitment of monocytes. An infection-inflammation-on-a-chip-model was developed. The dynamic equilibrium between migration, reversed-migration, and trapping processes determine the optimal number of neutrophils at a site and these neutrophils are continuously refreshed and responsive to the number of microbes [90]. They also found the important differences among migration counts, velocity, and directionality among neutrophils from 2 common mouse strains, rats, and humans by using this donut-shaped [91].

Based on the pressure balance zone, Yang et al. [26] developed an all-on-chip method for integrating the magnetic negative purification of neutrophils and chemotaxis assay from small blood volume samples (Fig. 7b). This device achieved a rapid sample-to-result neutrophil chemotaxis test in 25 min. At least 25% of the neutrophils from input whole blood sample effectively entered the docking structure and the purity of neutrophils was high by on-chip Giemsa staining. Gradient were generated based on the continuous laminar flow chemical mixing, and the flows were driven by the pressure difference from the different levels of inlet and outlet solution. The chemical gradient was established within a few minutes in the microfluidic channel and kept stable for at least 1 h. This device was used to test neutrophil chemotaxis in COPD sputum and the result showed a strong cell migration to the COPD sputum gradient.

Moussavi-Harami et al. [85] reported a “PI” channel for studying chemotaxis through a 3-D environment in the presents of dual chemotactic gradients and successfully performed neutrophil chemotaxis experiments with the whole blood sample in 2015. The PI channel included a chemoattractant containing gel which was perpendicular to a cell placement channel. The gel played as the 3D platform for cell migration from the cell placement channel. The gradients were generated in the gel to more closely mimic complex in vivo gradients. Moreover, the basement membrane-like substrate Matrigel was used in the gel region to maintain a stable gradient, without any manipulation of the device once the system settled. The migration of the neutrophil-like cell line PLB-985 in gradients of fMLP was tested using this device. The PLB-985 cells were observed to migrate in the presence of fMLP or LTB_4_ gradient with a velocity of 7.73 µm/min in single fMLP gradient and 8.01 µm/min in double fMLP gradient, while no migration was observed in control setups where no chemoattractant in the device. Furthermore, this device was used with heparinized whole blood and neutrophils were observed to migrate into the gel over 2.5 h with an average velocity of 3.7 µm/min without any neutrophil purification or capture steps.

Functional modification of selectins or antibodies is also a common method to purify neutrophil in microfluidic device. Agrawal et al. [84] firstly fabricated a microfluidic chip for neutrophil chemotaxis studies using P-selectin as the substrates for neutrophil isolation in 2008. Sachmann et al. [43] reported a KOALA platform that consisted of a lid and a base, and the device achieved neutrophil purification and chemotaxis on-chip within minutes in 2012 (Fig. 7c). It only required nanoliters of whole blood as the sample and a micropipette to operate. Neutrophils were specifically captured on the polystyrene surface functionalized by P-selectin. The device achieved ~ 80% capture efficiency of human primary neutrophils. The lid was used to house the reagents required to generate the gradient of chemoattractant. After capturing, the lid was placed onto the base, thereby allowing the chemoattractant in the lid to controllably diffuse into the microchannel to form a gradient of chemoattractant. By using this method, the difficulty of performing challenging experiments was significantly reduced. Compared with the traditional standard technology, this device achieved increased functionality and simplicity of operation. Moussavi-Harami et al. [86] reported a microfluidic device for simultaneous analysis of NETs and ROS. P-selectins were coated on the bottom of the channel to capture and purify neutrophils, and it achieved purify primary human neutrophil in less than 10 min from a few microliters of whole blood. The device showed the ability to distinct the neutrophil subsets (including POS production and NET formation) in different stimulants/inhibitors. The sample was still effectively used after storing for up to 8 h in the device.

Tay et al. [27] reported an integrated microfluidic device for single-step neutrophil sorting and chemotaxis study using a small blood volume. The purification of neutrophils from whole blood was based on the biomimetic cell margination and affinity-based capture. The separation efficiency of leukocyte (CD45+) was more than 80% and the device achieved 12-fold leukocyte enrichment at the side outlets. The captured neutrophils were then exposed in the diffusion-based chemotactic gradients environment to initiate chemotaxis. The result showed significant reduce of TNF-α and glucose-treated neutrophils migration toward fMLP (≈ 45%) compared with healthy neutrophils migration (≈ 66.2%), while exhibited a significant decrease in cell migration. A decrease in chemotaxis velocity was also observed in TNF-α treated neutrophils (≈ 5.22 ± 0.37 µm min − 1) when compared to healthy neutrophils (≈ 6.27 ± 0.59 µm min^−1^). In addition, distinct neutrophil chemotaxis suppression was observed in vitro inflamed blood sample within few minutes. Therefore, the method can be used in rapid assessment of neutrophil functions.

#### Combined with the analysis unit

**Fig. 8:**
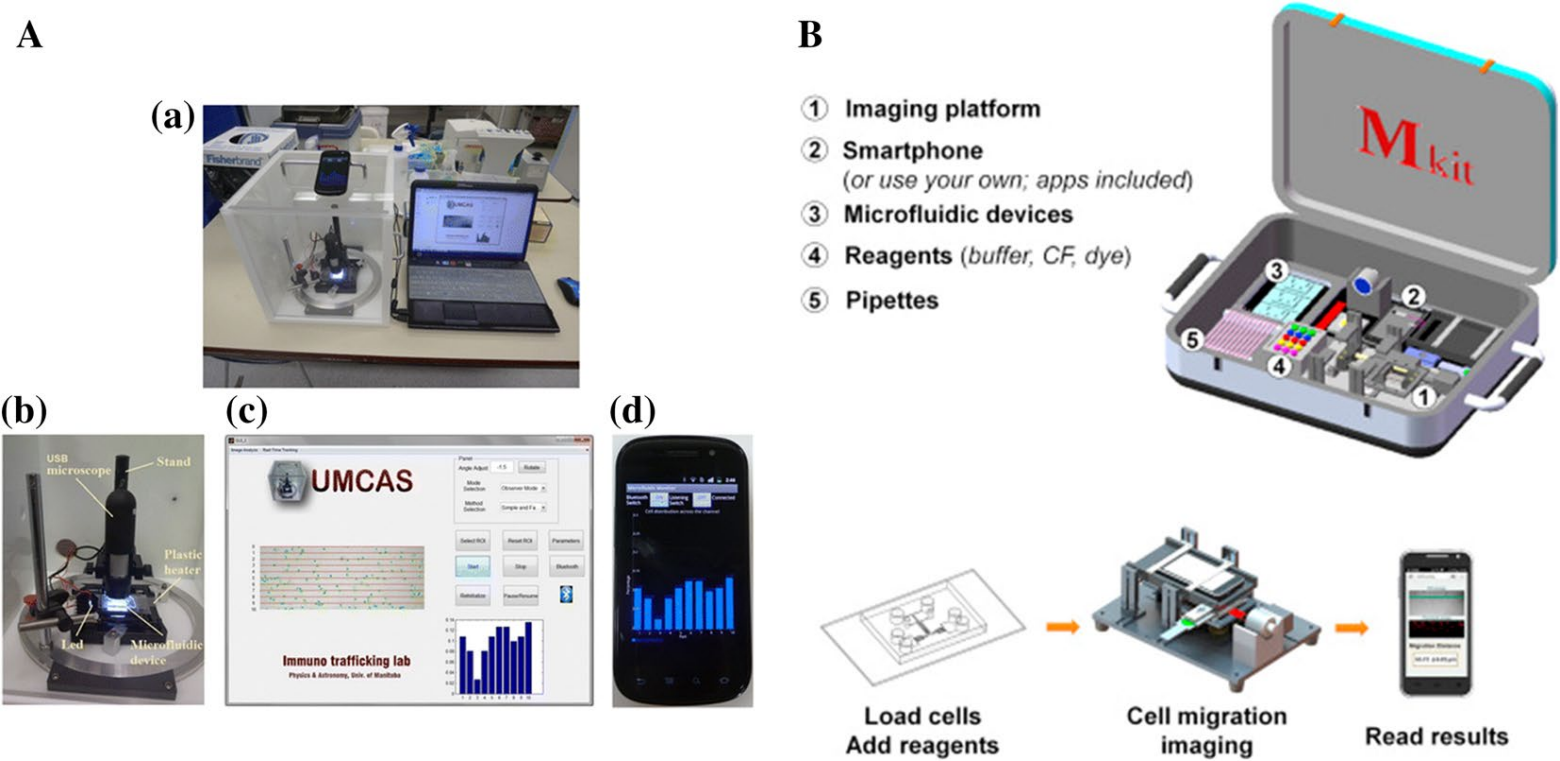
Examples of integrated neutrophil chemotaxis devices combined with analysis units. **A** The USB microscope-based microfluidic chemotaxis system (UMCAS). This system contains microfluidic device, live cell imaging, control and analysis units. (Figure reproduced from Ref. [95]); **B** components and operation flow of the Mkit. Reprinted from Ref. [48], Copyright (2017), with permission from Elsevier

The real-time diagnosis and portability of instruments have become more and more important as medical health is gradually valued by people. Therefore, it is emerging research filed to make real-time detection and analysis systems by combing the microfluidic device with the analysis unit. Wu et al. [95] developed a compact USB microscope-based microfluidic chemotaxis analysis system (UMCAS) which integrated microfluidic devices, live cell imaging, environmental control and data analysis (Fig. 8A). It provided a platform for rapid microfluidic neutrophil chemotaxis experiments with real-time data analysis and wireless remote data monitoring.

Mobile sensing based on the integration of microfluidic device and smartphone (MS^2^) technology is a fast developing filed in the recent years. It has been used in a wide range of application, such as biochemical detection and disease diagnosis [96]. Compared with the traditional platforms, MS^2^ technology provides significant advantages in terms of test speed and control, low cost, ease-of-operation and data management. Recently, Yang et al. developed a MS^2^-based cell functional assay for testing cell migration (the Mkit) based on their previous work [26, 48] (Fig. 8B). This system combined microfluidic chip, a smartphone-based imaging platform, the phone apps for image capturing and data analysis, and a set of reagent and accessories for performing the cell migration assay together. With the optical components, the system achieved ~ 3 µm resolution which was adequate for imaging of many types of cells. Cells in the images could be identified by the image processing and data analysis app, and the cell migration distance along the gradient direction could be calculated as a measure of chemotaxis. This system successfully measured chemotaxis from purified neutrophil, cancer cells. Furthermore, neutrophil chemotaxis was tested from whole blood sample and clinical samples from chronic obstructive pulmonary disease patient.

This type of system that integrated detection and analysis units greatly reduces the requirement of operators. However, it is still in the initial stage of research. A series of problems needed to be solved, such as low resolution, poor stability, and few analytical functions.

### Applications

With the gradual maturity of the technology, microfluidic neutrophil chemotaxis devices have more and more clinical application.

**Fig. 9:**
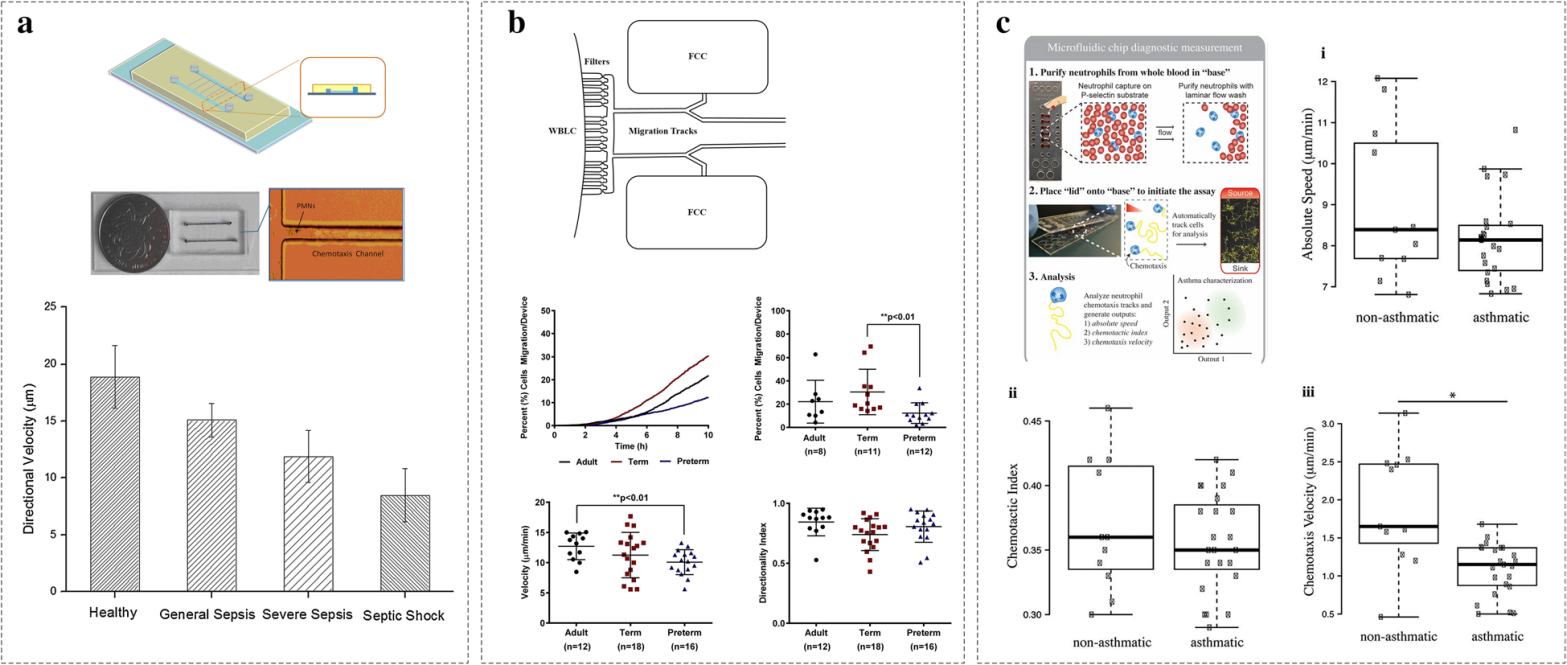
Examples of microfluidic neutrophil chemotaxis devices in clinical applications. **a** The overview of the gel-based neutrophil chemotaxis device. The gel channel (100 µm high) was used for collagen loading. The cell channel (50 µm high) was used for cell loading. The linear LPS concentration gradient was generated in five migration channels (50 µm high). The directional velocities of healthy, general sepsis, severe sepsis, and septic shock groups were decreased with the seriousness of sepsis. Reprinted from Ref. [33], Copyright (2016), with permission from Elsevier; **b** the donut-shape neutrophil chemotaxis assay and characterization of neutrophil chemotaxis of adults, term neonates, and preterm neonates. Reprinted from Ref. [92] Copyright (2017), with permission from Elsevier; **c** the microfluidic method for phenotyping asthma patients. For asthmatic and nonasthmatic patients, no statistically significant difference in neutrophil migration speed (i) and chemotactic index (ii); for asthmatic patients (n = 23), neutrophil chemotaxis velocity (iii) is lower than nonasthmatic patients (n = 11). *P = 0.02. (Figure reproduced from Ref. [39])

For example, combined microgroove structure and hydrogel, Lu et al. [33] fabricated a channel-microgrooves-channel microfluidic chip to measure neutrophil chemotaxis and assess the severity and prognosis of sepsis (Fig. 9a). Two main channels were filled with polymorphonuclear neutrophils (PMNs) and a collagen gel that was spiked with LPS, respectively. The collagen gel containing the LPS was used to generate a concentration gradient in microgrooves and reduce the shear stress acting on the PMNs. PMNs moved in the microgrooves towards gel channel driven by the LPS concentration gradient. Neutrophils of 32 sepsis patients were divided into three groups according to the seriousness, and 12 healthy individuals served as controls. Results showed that neutrophil chemotaxis was significantly decreased following the seriousness of sepsis.

Raymond et al. [92] provided the first description of neutrophil chemotaxis and transcriptomics from whole blood of human term and preterm neonates, as well as young adults using the donut-shape microfluidic device (Fig. 9b). Ex vivo whole blood chemotaxis was measured to the fMLP for term neonates (n = 20), preterm neonates (n = 20), and young adults (n = 15). Neutrophils from preterm neonates migrated in fewer numbers compared with term neonates (preterm 12.3%, term 30.5%), and at a reduced velocity compared with young adults (preterm 10.1 µm/min, adult 12.7 µm/min). Ex vivo spontaneous neutrophil migration, neutrophil transcriptomics, and cytokine production in the presence and absence of LPS were measured directly from whole blood of adults, term neonates, and preterm neonates to understand the response of human neonatal neutrophils to toll-like receptor (TLR4) stimulation [93]. The results showed significantly fewer spontaneously migration neutrophils of preterm neonates at baseline, and compared to adults, both term and preterm neonates had decreased neutrophil velocity. In the presence of LPS stimulation, the number of spontaneously migrating neutrophils of preterm neonates was reduced compared to term neonates and adults.

Based on the KOALA platform, Sackmann et al. [39] developed a microfluidic-based handheld diagnostic device to discriminate asthma from allergic rhinitis based on the patient’s neutrophil chemotactic function (Fig. 9c). It was determined that neutrophils from asthmatic patients (n = 23) migrated significantly slowly toward the chemoattractant compared with nonasthmatic patients(n = 11) (P = 0.02). The device needed low requirements of sample volume and the diffusion of laminar fluidics provided precise control microenvironment of cells.

## Conclusion

In summary, we reviewed the recent developments in microfluidic-based neutrophil chemotaxis studies with different gradients generation methods. The integrated microfluidic devices based on various functions were also discussed. By taking the advantages of microfluidics, microfluidic devices have been a popular tool for cell chemotaxis studies. The devices can provide stable, controlled, and complex profiles gradients and a shear-free environment for neutrophils by changing the materials and the structure of the devices. Meanwhile, the studies of integrated system have been increased and had the potential for clinical applications, especially some researches aimed at how to achieve chemotaxis assay directly from the whole blood. However, there are still some challenging issues of microfluidic-based neutrophil studies. The major challenges are to precisely control the concentration gradients of chemotactic agents and to reduce the impact of the fluid shear forces on neutrophils. The control of other elements of the microenvironment, such as pH and temperature, is still unstable. The integration of the devices is low with lacking of the integration of signal acquisition and processing modules. Automated analytical systems are needed for clinical and commercial application. The analysis and information extraction of the results obtained from neutrophil chemotaxis assays and further modeling of the neutrophil chemotaxis process and mechanism analysis are issues to be considered.

With the development of microfluidic technology, neutrophil chemotaxis studies will be developed in the direction of multi-functions, easy-operation, and integration. Microfluidic technology will become a useful technical tool to study the mechanism of neutrophil chemotaxis and microfluidic-based neutrophil chemotaxis devices will have more application not only in basic researches but also in clinical applications.

## Data Availability

Not applicable.

## Abbreviations

bMFA: Bioinspired microfluidic assay
µ-TAS: Micro-total analysis system
PDMS: Polydimethlsiloxane
µC^3^: Microfluidic competitive chemotaxis-chip
CGG: Concentration gradient generator
MtLN3: Metastatic rat mammary adenocarcinoma cells
µFCD: Microfluidic chemotaxis device
TEM: Transendothelial migration
UCB: Umbilical cord blood
WBLC: Whole blood loading chamber
FCCs: Focal chemotaxis chambers
UMCAS: USB microscope-based microfluidic chemotaxis analysis system
PMNs: Polymorphonuclear neutrophils
TLR4: Toll-like receptor
MSCs: Mesenchymal stem cells
IL-6: Interleukin-6
PDGF: Platelet derived growth factor
ECM: Extracellular matrix
DMSO: Dimethyl sulfoxide
PMN: Polymorphonuclear cells
TSP: Thrombospondin
RA: Retinoic acid
l-AA: l-ascorbic acid
GPCRs: G protein-coupled receptors
PMMA: Polymethyl methacrylate
PC: Polycarbonate
MS^2^: Microfluidic device and smartphone
